# Supplementary material for: Signaling mechanisms in renal compensatory hypertrophy revealed by multi-omics
Source: Nat Commun. 2023 Jun 16;14:3481. doi: 10.1038/s41467-023-38958-9 (PMC10276015; doi:10.1038/s41467-023-38958-9)
Supplement: Supplementary file 1 — Supplementary Information [file 41467_2023_38958_MOESM1_ESM.pdf]

NCOMMS-22-37275A

Signaling Mechanisms in Renal Compensatory Hypertrophy Revealed by Multi-Omics

Supplementary information

See also https://esbl.nhlbi.nih.gov/Databases/UNx-Supp/

Index:

Pages 2-3. Supplementary Methods: Statistical methods used
Page 4. Supplementary Methods: Deconvolution of bulk RNA-seq data.
Pages 5-28. Supplementary Figures with Legends
Pages 29-31 Supplementary Tables 1-4 with Legends
Pages 32-36 List of Supplementary Data Available at https://esbl.nhlbi.nih.gov/Databases/UNx-Supp/
Pages 37-38 Supplementary Discussion 1
Pages 39 Supplementary Discussion 2
Pages 40-42 Supplemental References

Supplementary methods: Statistical methods used

Data Type	Measure	Metrics	Criterion
Body weight and Kidney weight (**Figs. 1b, Figs. 10b,h,i, Supplementary Figs. 1a** and **b**, **Supplementary Figs 7c,g** and **h**)	Kidney weight (mg), Body weight (g)	P value (unpaired T-test)	P < 0.05
Quantitative histology for kidney (**Fig. 1d, Fig. 10c, Supplementary Figs. 1a** and **b**)	Length (mm)	P value (unpaired T-test)	P < 0.05
Quantitative immunohistochemistry in microdissected tubules (**Fig. 1e, Fig. 10e** and **j, Supplementary Fig. 1d, Supplementary Fig.7i**)	Cell volume (um^3), Outer diameter (um), Count/length (count/mm)	P value (unpaired T-test)	P < 0.05
Semiquantitative immunoblotting of kidney (**Supplementary Figs. 6c** and **d**)	Normalized band density	P value (unpaired T-test)	P < 0.05
ATAC-Seq peaks UNx vs Sham (**Fig. 3a**)	log_2(UNx/Sham) values	FDR (Benjamini and Hochberg method)	FDR < 0.05
Distributions of target peaks/genes/proteins (**Fig. 3f, 6c, 7b, 8b, Supplementary Figs. 2e, 3b, 6a**)	log_2(UNx/Sham) values	Pearson's Chi-squared test with Yates' continuity correction	P < 0.05
RNA-Seq: UNx vs Sham (**Fig. 4a, 5a,** and **6a, Supplementary Fig. 4c**)	log_2(UNx/Sham) values	Adjusted P-value (Benjamini and Hochberg method)	P_{adj}<0.05
Proteomics: UNx vs Sham (**Figs. 7a, 7f, 8a**)	log_2(UNx/Sham) values	P value (unpaired T-test)	P < 0.1
Semiquantitative Immunocytochemistry of kidney tissues (**Fig. 5d**)	Positively stained area	P value (unpaired T-test)	P < 0.05

Correlation between RNA-seq data and ATAC-seq data in $\log_2$(UNx/Sham) (**Figs. 4e** and **f, Supplementary Figs 3d**) Correlation between RNA- seq data and Proteomics data (**Figs. 7c** and **d, Supplementary Fig. 6b**)	Scatter plot	P value (Pearson correlation)	$P < 0.05$
Quantitative RT-PCR (**Figs. 10a, g** and **Supplementary Fig 7b**)	gene expression normalized to β-actin	P value (unaired T- test)	$P < 0.05$
Free fatty acid, triglycerides, phospholipids and total cholesterol concentration analysis(**Figs. 9c** and **e**)	ug/mg kidney	P value (unpaired T-test)	$P < 0.05$

Supplementary methods: **Deconvolution of bulk RNA-seq data**

Genes exhibiting differential expression (FDR-adjusted *p*-values < 0.1) were selected from the UNx vs. Sham RNA-seq data at the 24-hour time point. These genes were further filtered by comparison to a snRNA-seq data set from a mouse ischemic-renal injury model[1]. in short, genes classified with the annotations of “Healthy S1” and “Injured/Severe injured PT” in the snRNA-seq data set were selected from the list of differentially expressed genes in our data set and used for this deconvolution analysis (**Supplementary Discussion 2**).

Supplementary Figures

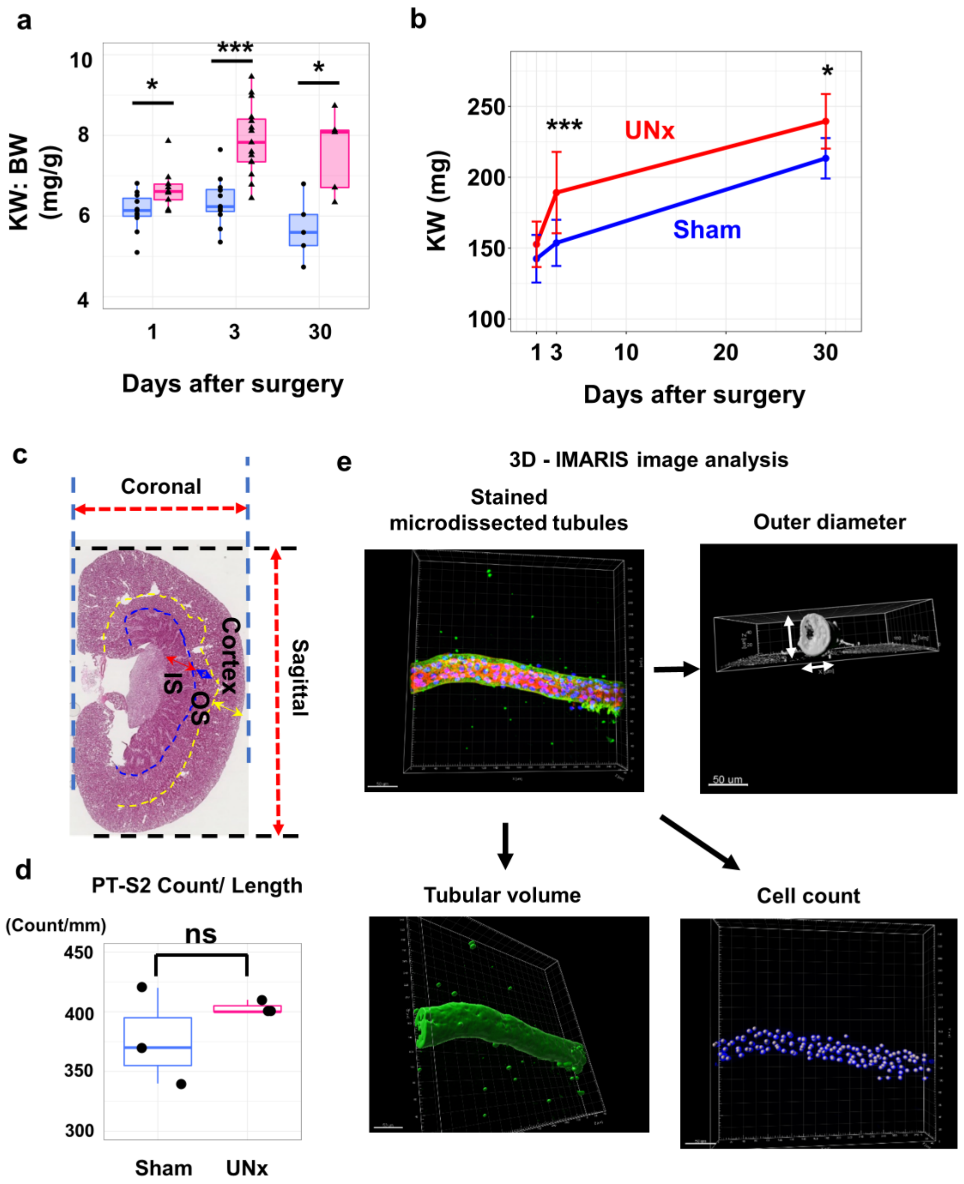

Supplementary Figure 1. Morphological evaluation of compensatory hypertrophy after unilateral nephrectomy (UNx).

(a) Box plot of kidney weight over body weight at 1,3, and 30 days after surgery. (*n* = 11 for Sham-day 1, *n* = 12 for Sham-day 3, *n* = 5 for Sham-day 30, *n* = 9 for UNx-day 1, *n* = 15 for UNx-day 3, *n* = 5 for UNx-day 30). *$p < 0.05$, ***$p < 0.001$ (Day1: p = 0.0207, Day3: p = 0.00002, Day30: p = 0.011, unpaired, two-sided student T- test).

(b) Time course indicating the kidney weight (KW) at 1, 3, and 30 days after surgery (*n* = 11 for Sham-day1, *n* = 12 for Sham-day3, *n* = 5 for Sham-day30, *n* = 9 for UNx-day1, *n* = 15 for UNx-day3, *n* = 5 for UNx-day30). Data are presented as mean ± SD. *$p < 0.05$, ***$p < 0.001$ (Day1: p = 0.1857, Day3: p = 0.0004, Day30: p = 0.00105, unpaired, two-sided student T- test).

(c) Representative image of hematoxylin and eosin (H&E) stained kidney showing coronal length, sagittal length, and regions of cortex , outer stripe of outer medulla and inner stripe of outer medulla. The arcuate arteries are used as a border of the renal cortex and renal medulla.

(d) Cell count per unit length in proximal tubule S2 region was not changed between Sham and UNx. (*n* = 3 for each group). Data are presented as mean ± SD.

(e) 3D analysis of IF-stained microdissected tubules by IMARIS software.

Stained microdissected tubules (AQP1, green; F-actin, red; DAPI labeling of nuclei, blue) were used for the calculation of tubular outer diameter (Top right), tubular volume (Bottom left), and cell count (Bottom right) by IMARIS software (v9.9.1; Bitplane, Zurich, Switzerland)

Box-and-whisker plots represent median and 25th and 75th percentiles—interquartile range; IQR—and whiskers extend to maximum and minimum values.

*$p < 0.05$, **$p < 0.01$, ***$p < 0.001$ (unpaired, two-sided student T- test).

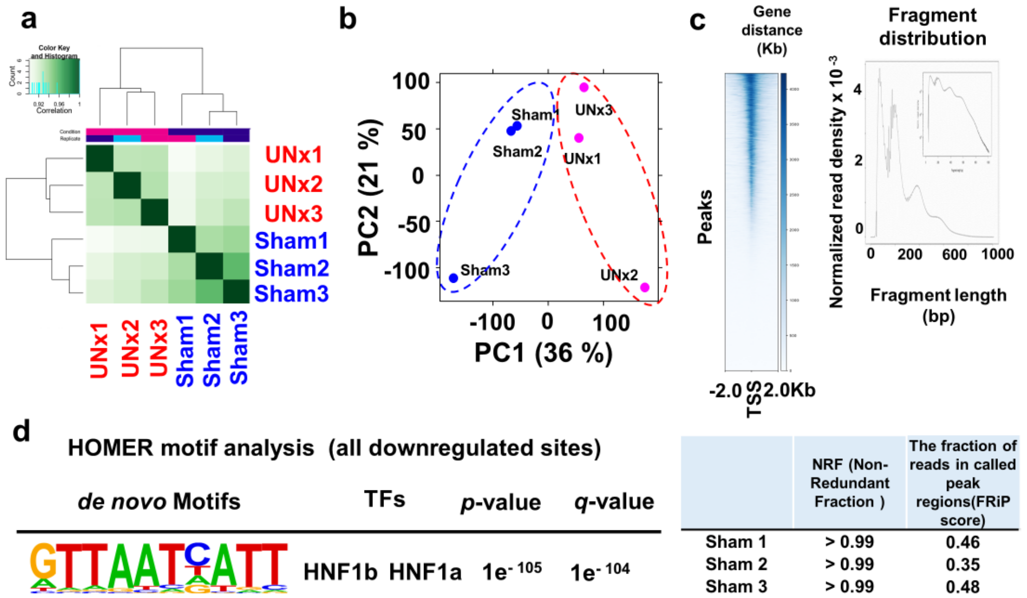

d

HOMER motif analysis (all downregulated sites)

de novo Motifs	TFs		*p*-value	*q*-value
GTTAATCATT	HNF1b	HNF1a	1e-105	1e-104
CTTGCCAA	NFIX	NFIA	1e-14	1.11e-14

	NRF (Non-Redundant Fraction)	The fraction of reads in called peak regions(FRiP score)
Sham 1	> 0.99	0.46
Sham 2	> 0.99	0.35
Sham 3	> 0.99	0.48
UNx 1	> 0.99	0.48
UNx 2	> 0.99	0.52
UNx 3	> 0.99	0.64

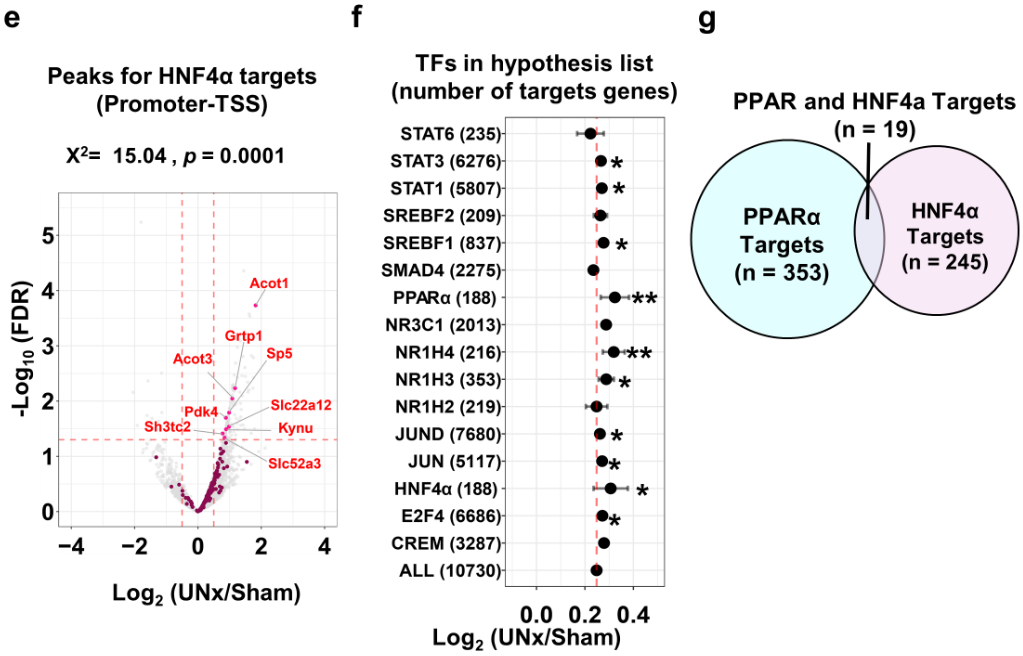

Supplementary Figure 2. Difference of chromatin accessibility of PTs between Sham and UNx at the 24 hour timepoint.

(a) Heatmap clustering of Pearson correlation coefficient showing the correlation between the replicates of the conditions, namely, Sham and UNx sample replicates.

(b) Principal component analysis (PCA) of Sham and UNx sample replicates. Projection of samples along the first two principal components denoted by dotted circles. PC1 and PC2 describe 36% and 21% of the variability, respectively.

(c) Quality control for ATAC seq data. (Upper Left) Heatmap of ATAC-seq read densities at +/- 2 kb around annotated TSS. (Upper Right) Fragment size distribution plot. Enrichment around 100 and 200 bp is indicates nucleosome-free and mono-nucleosome-bound fragments. (Lower) Table summarizing non-redundant fraction (NRF), the fraction of reads in called peak regions (FRiP score), and read depth after removal of duplicate.

(d) HOMER analysis identifies the enriched TF binding motifs in chromatin regions that are significantly less accessible in UNx applying cumulative hypergeometric distribution adjusted for multiple testing with the Benjamini-Hochberg method.

(e) Volcano plots indicating chromatin accessibility at promoter regions near annotated TSS. Magenta-colored points indicate HNF4α target regions. Peaks with significant changes (FDR <0.05) were annotated with associated gene names. Target genes for PPARα are listed in **Supplementary Data 7**. p-values represent the likelihood of the distribution of peaks using Chi-squared analysis and threshold criteria of $|\log2(UNx/Sham)| > 0.5$ and FDR < 0.05.

(f) Target gene set analysis for TFs listed in **Supplementary Table 1** at 24 hours after UNx using peaks detected in promoter-TSS regions. Log_2(UNx/Sham) values were plotted for members of curated target gene sets. *p < 0.05, **p < 0.01 (un-paired, two-tailed Student's t-test, p values are provided in **Supplementary Data 8**), (n = 3 for Sham, n = 3 for UNx). Error bars indicate 95% confidence interval allowing comparison with the average of log_2 (UNx/Sham) for all identified regions (vertical dashed line). The measure of center for the error bars is the averages of log_2 ratios (UNx/Sham) of peak concentrations (in ATAC-seq) for TF-target gene sets. TF, transcription factor

(g) Venn diagram of PPARα target genes (blue circle) and HNF4α target genes (red cercle). PPARα and HNF4α share 19 target genes listed in **Supplementary Data 7**.

UNx, unilateral nephrectomy *p < 0.05, **p < 0.01.

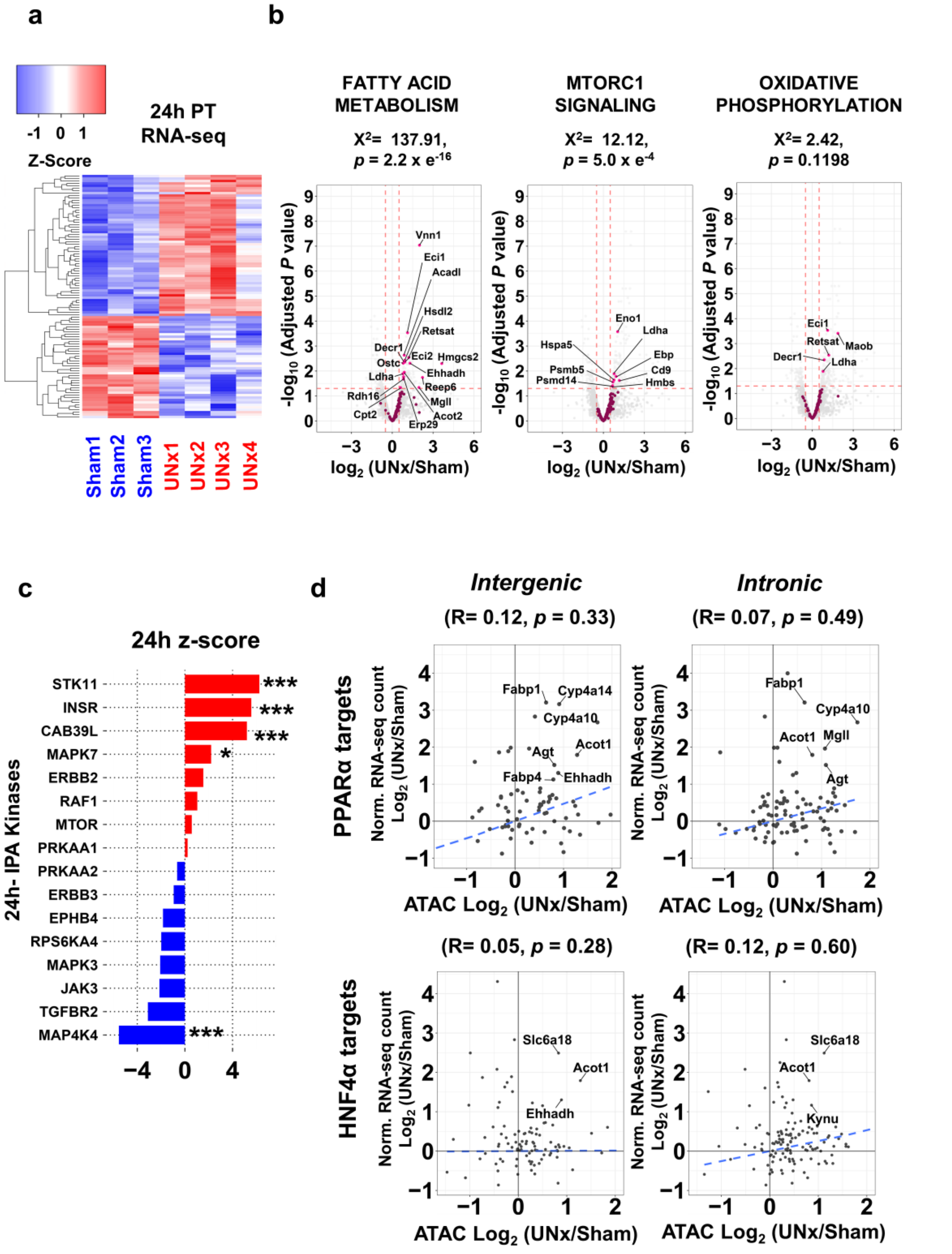

Supplementary Figure 3. Transcriptome differences in proximal tubule S1 segment between Sham and UNx at the 24 hour timepoint.

(a) Heatmap of normalized RNA-seq read counts (Log_{10} transformed, scaled and centered) from proximal tubule of Sham (n = 3) and UNx (n = 4) at the 24h timepoint. Data shown are the top 100 most variable genes (based on coefficient of variance) with total minimum counts greater than 100.

(b) Volcano plots of gene sets annotated with the GSEA terms "FATTY_ACID_METABOLISM", "MTORC1_SIGNALING" and "OXIDATIVE_PHOSPHORYLATION". These terms were identified as upregulated using GSEA on the 24h RNA-seq dataset from microdissected proximal tubules. Magenta-colored points indicate genes annotated as "FATTY_ACID_METABOLISM" (Left), "MTORC1_SIGNALING" (Center) and "OXIDATIVE_PHOSPHORYLATION" (Right) in GSEA. Genes with statistically significant changes between UNx and Sham were annotated with gene names. Significant differential expression was determined using thresholds of $p_{adj} < 0.05$ (Benjamini and Hochberg method) and $\log_2$ (UNx/Sham) gt. 0.5 or lt. -0.5. GSEA annotations are provided on the GSEA website (https://www.gsea-msigdb.org/gsea/msigdb/human/genesets.jsp?collection=H). *p*-values represent the likelihood of the distribution of proteins using Chi-squared analysis.

(c) Prediction of upstream regulatory kinases determined using Ingenuity Pathway Analysis (IPA). Predictions were performed using differentially expressed genes in UNx vs. Sham treatments at 24 hours. *$p < 0.05$, **$p < 0.01$, ***$p < 0.001$. (Right-tailed Fisher's Exact Test, p values are provided in **Supplementary Data 10**)

(d) Correlations (Pearson's correlation) between gene expression and chromatin accessibility at Intergenic regions (left) and at intronic regions (Right) for UNx vs. Sham treatments. (Top) PPARα target genes. (Bottom) HNF4α target genes. The *x*-axes indicate $\log_2$(UNx/Sham) of normalized peak read concentration from ATAC-seq data. The *y*-axes indicates $\log_2$(UNx/Sham) of normalized read counts from RNA-seq data. Significant correlation was assessed with Pearson's product moment correlation coefficient using the stat_cor function (method = Pearson, two-sided) in R.

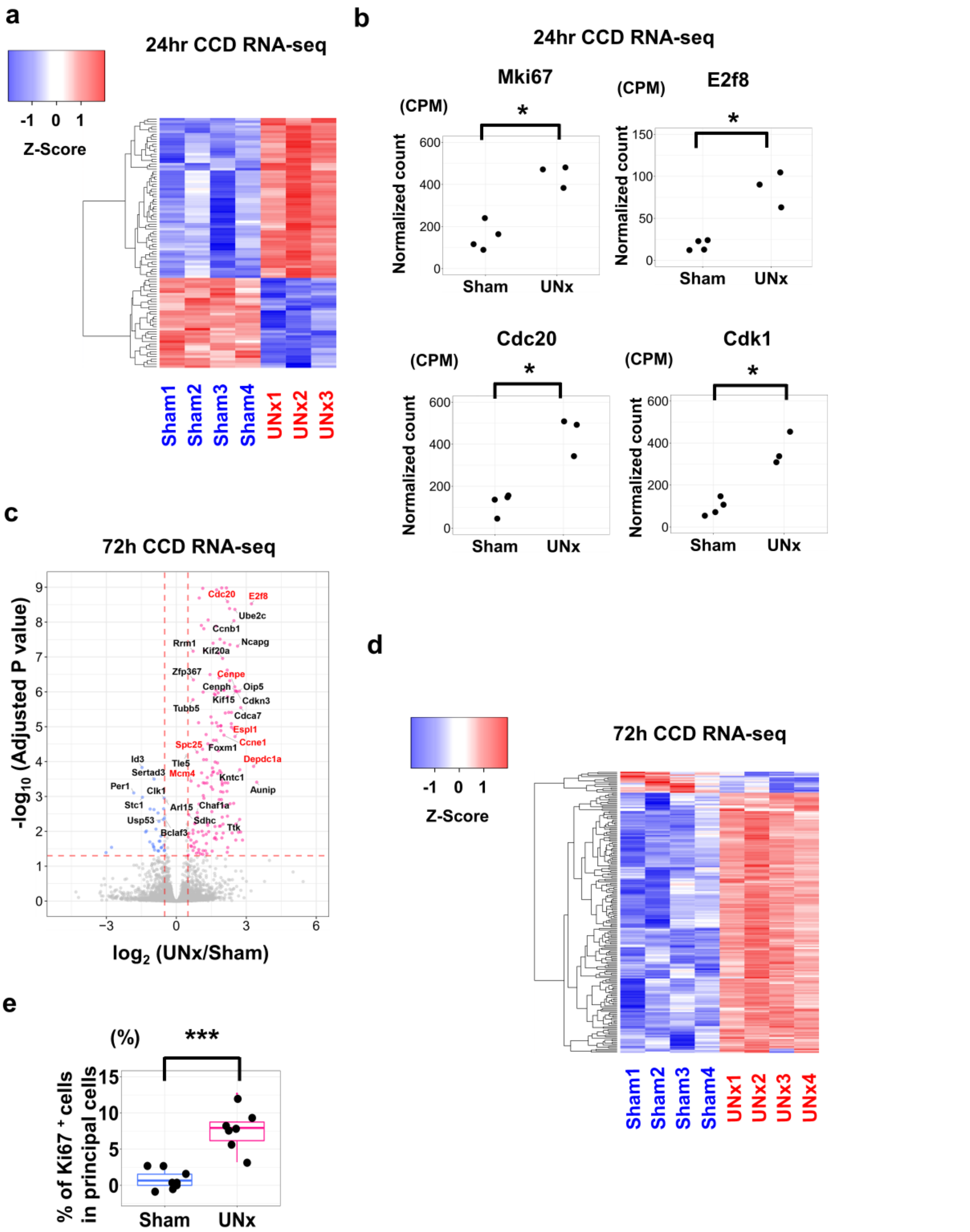

Supplementary Figure 4. Transcriptome difference in cortical collecting ducts (CCD) between Sham and UNx.

(a) Heatmap of normalized RNA-seq counts (Log_{10} transformed, scaled and centered) from cortical collecting duct (CCD) of Sham (*n* = 4) and UNx (*n* = 3) at the 24 h timepoint. Data shown are the top 100 most variable genes (based on coefficient of variance) with total minimum counts greater than 100.

(b) Normalized read counts of selected cell-cycle related genes in the CCD from Sham and UNx at the 24 h timepoint. ***p < 0.001, unpaired, two-sided student T- test. (Mki67: p = 0.000015 E2f8: p = 0.000001, Cdc20: p = 0.000005, Cdk1: p = 0.0000001)

(c) Volcano plot showing UNx versus Sham for microdissected CCDs at the 72h timepoint. Red dots indicate significantly increased genes in UNx ($P_{adj} < 0.05$ (Benjamini and Hochberg method) and log_2 (UNx/Sham) > 0.5), blue dots indicate significantly downregulated genes in UNx ($P_{adj} < 0.05$ and log_2 (UNx/Sham) < -0.5).

(d) Heatmap of normalized RNA-seq counts (Log_{10} transformed, scaled and centered) from CCD of Sham (*n* = 4) and UNx (*n* = 4) at the 72h timepoint. Data shown are the top 100 most variable genes (based on coefficient of variance) with total minimum counts greater than 100.

(e) percentage of Ki67-positive cells. Scale bar indicates 200 μm. Difference was found to be significant using a two-tailed Student's *t*-test, ***p < 0.001 (p = 0.00061). Data are representative of multiple independent biological replicates (Sham; *n* = 8, UNx; *n* = 7). Box-

and-whisker plots represent median and 25th and 75th percentiles—interquartile range; IQR—and whiskers extend to maximum and minimum values.

*$p < 0.05$, **$p < 0.01$, ***$p < 0.001$.

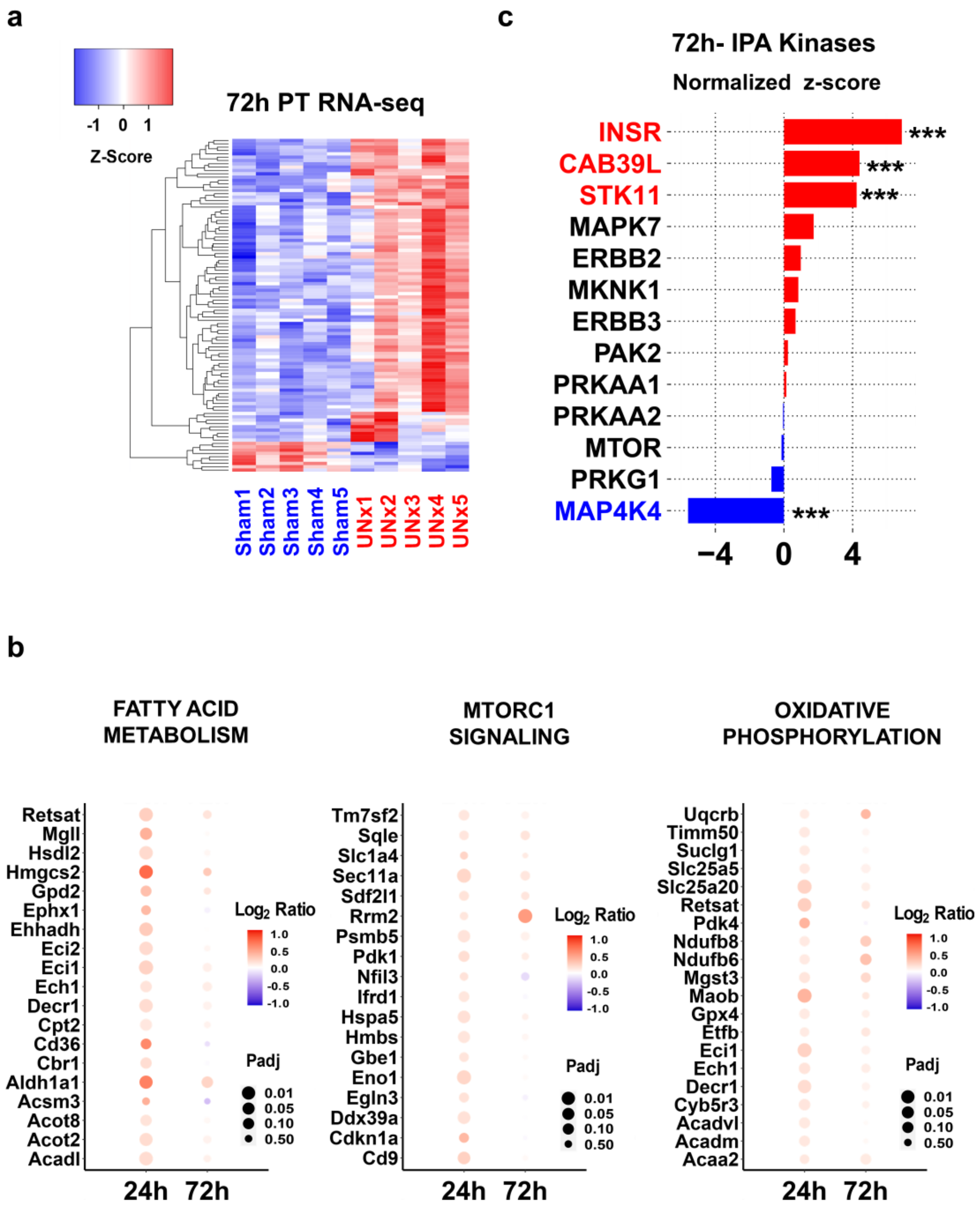

Supplementary Figure 5. Transcriptome difference in the proximal tubule S1 segment between Sham and UNx at the 72 hour timepoint.

(a) Heatmap for normalized RNA-seq counts (Log_{10} transformed, scaled and centered) from proximal tubule of Sham (*n* = 5) and UNx (*n* = 5) at the 72 h timepoint. Data shown are the top 100 most variable genes (based on coefficient of variance) with total minimum counts greater than 100.

(b) Bubble plots showing differential changes in gene expressions between the 24 hour and 72 hour time points. Categories refer to the GSEA terms “FATTY ACID METABOLISM”, “MTORC1_SIGNALING”, and “OXIDATIVE_PHOSPHORYLATION”. Adjusted *p*-values (Benjamini and Hochberg method) are visualized by circle sizes (larger circles indicate smaller *p*-values), and log_2 ratios are visualized by color.

(c) Prediction of upstream regulatory kinases determined using Ingenuity Pathway Analysis (IPA). Proteins with normalized z-scores greater than 2 are colored in red, and those with normalized z-scores less than -2 are colored in blue. The statistical significance (*p*-values) returned in analyses is determined by calculating the extent to which the upstream regulators associated with dataset deviate from what was expected by chance alone in Fisher’s exact test. $^*p < 0.05$, $^{**}p < 0.01$, $^{***}p < 0.001$.

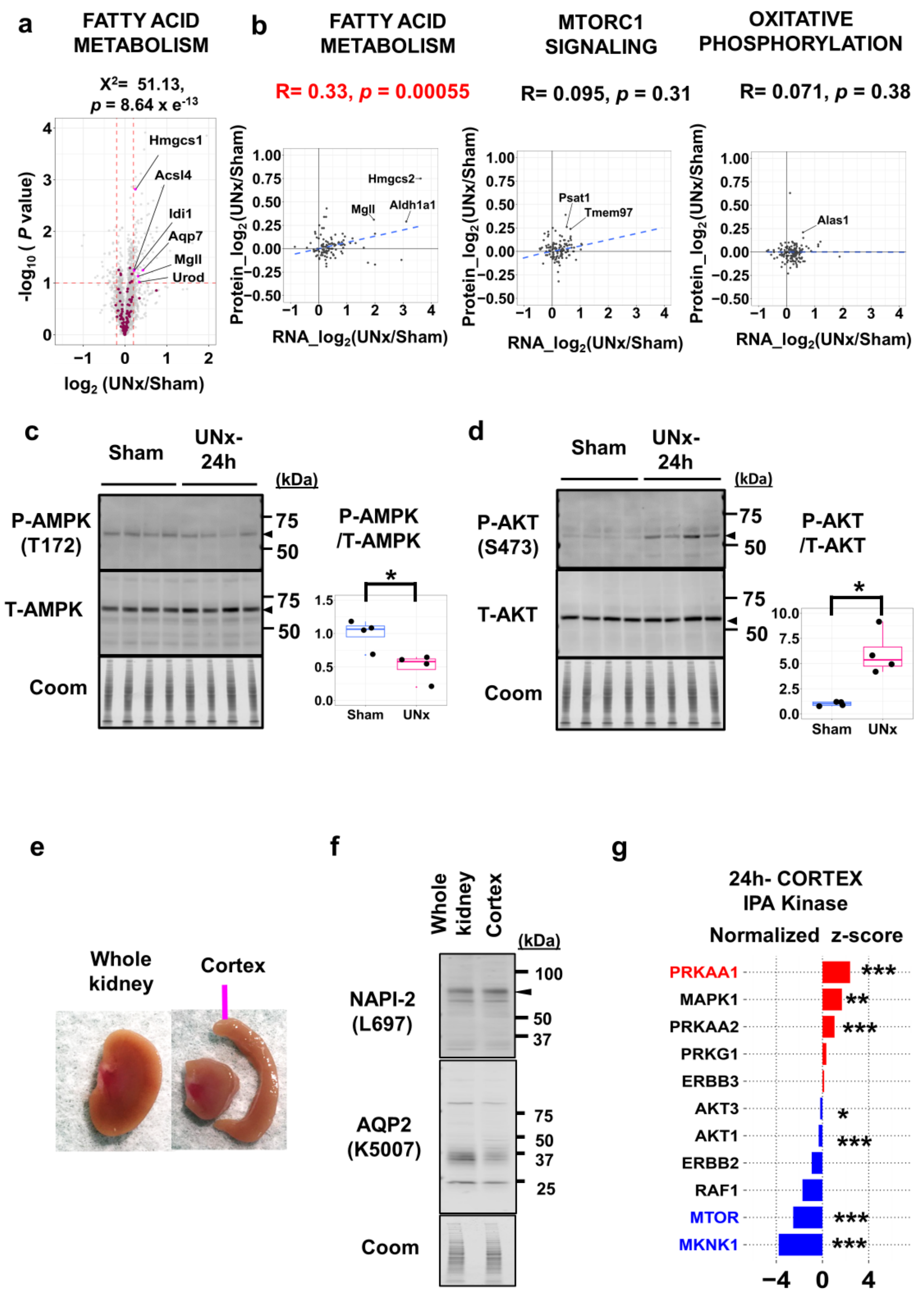

Supplemental Figure 6. Quantitative proteomics of whole kidneys and kidney cortex from Sham and UNx at the 24 h timepoint.

(a) Volcano plot of protein sets annotated with the GSEA terms "FATTY ACID METABOLISM" (indicated by magenta-colored points). Proteins with significant changes between UNx and Sham were annotated with protein names. Significant differential expression was determined using thresholds of $p < 0.1$ (unpaired, two-sided student T- test) and $\log_2$ (UNx/Sham) gt. 0.2 or lt. -0.2. GSEA target genes are provided in the GSEA website (https://www.gsea-msigdb.org/gsea/msigdb/human/genesets.jsp?collection=H). The statistical significance (p-values) returned in analyses is determined by Chi-squared analysis.

(b) Scatter plots of RNA-seq count ratios vs. protein abundance ratios for the UNx and Sham treatments using sets of genes annotated with the indicated GSEA terms. Axes are Log_2-scaled. Significant correlation was assessed with Pearson's product moment correlation coefficient using the stat_cor function (method = Pearson, two-sided) in R.

(c) (Left) immunoblot of total AMPK (T-AMPK) and phospho-Thr172 AMPK (P-AMPK) in Sham and UNx kidneys at the 24 hour time point. (Right) densitometry of the mean P-AMPK/T-AMPK ($n = 4$). UNx was found to exhibit a significantly lower ratio with a *p*-value threshold of < 0.05 ($p = 0.017$, unpaired, two-sided student T- test).

(d) (Left) immunoblot of total AKT (T-AKT) and phospho-Ser473 (P-AKT) in Sham and UNx kidney at the 24 hour timepoint. (Right) densitometry of the mean P-AKT/T-AKT (n = 4). UNx

was found to exhibit a significantly higher ratio using unpaired-student *t*-test with a *p*-value threshold of < 0.05. Coom, Coomassie Blue staining. ($p = 0.019$, unpaired, two-sided student T-test).

(e) Representative images of whole kidney (left) and kidney cortex (right)**.** Kidney cortex was dissected along the corticomedullary junction

(f) Western blot of NaPi-2 and AQP2 confirming the enrichment of the proximal tubule fraction in kidney. NaPi-2 (proximal type 2 Na-dependent phosphate transporter, L697), selectively expressed in proximal tubule, is used for showing the extent to which proximal tubule fraction is enriched in the sample. AQP2, which does not express in proximal tubule, is used for showing the extent to which how collecting duct fraction is de-enriched in the sample. Antibody targets are indicated on the *x*-axis. Coom is used as a loading control.

(g) Prediction of upstream regulatory kinases determined using Ingenuity Pathway Analysis (IPA) at 24 hours using kidney cortex. Proteins with normalized z-scores greater than 2 are colored in red, and those with normalized z-scores less than -2 are colored in blue. The statistical significance (*p*-values) returned in analyses is determined by calculating the extent to which the upstream regulators associated with dataset deviate from what was expected by

chance alone in Fisher's exact test. *$p < 0.05$, **$p < 0.01$, ***$p < 0.001$. p values are provided in **Supplementary Data 10.**

Box-and-whisker plots represent median and 25th and 75th percentiles—interquartile range; IQR—and whiskers extend to maximum and minimum values.

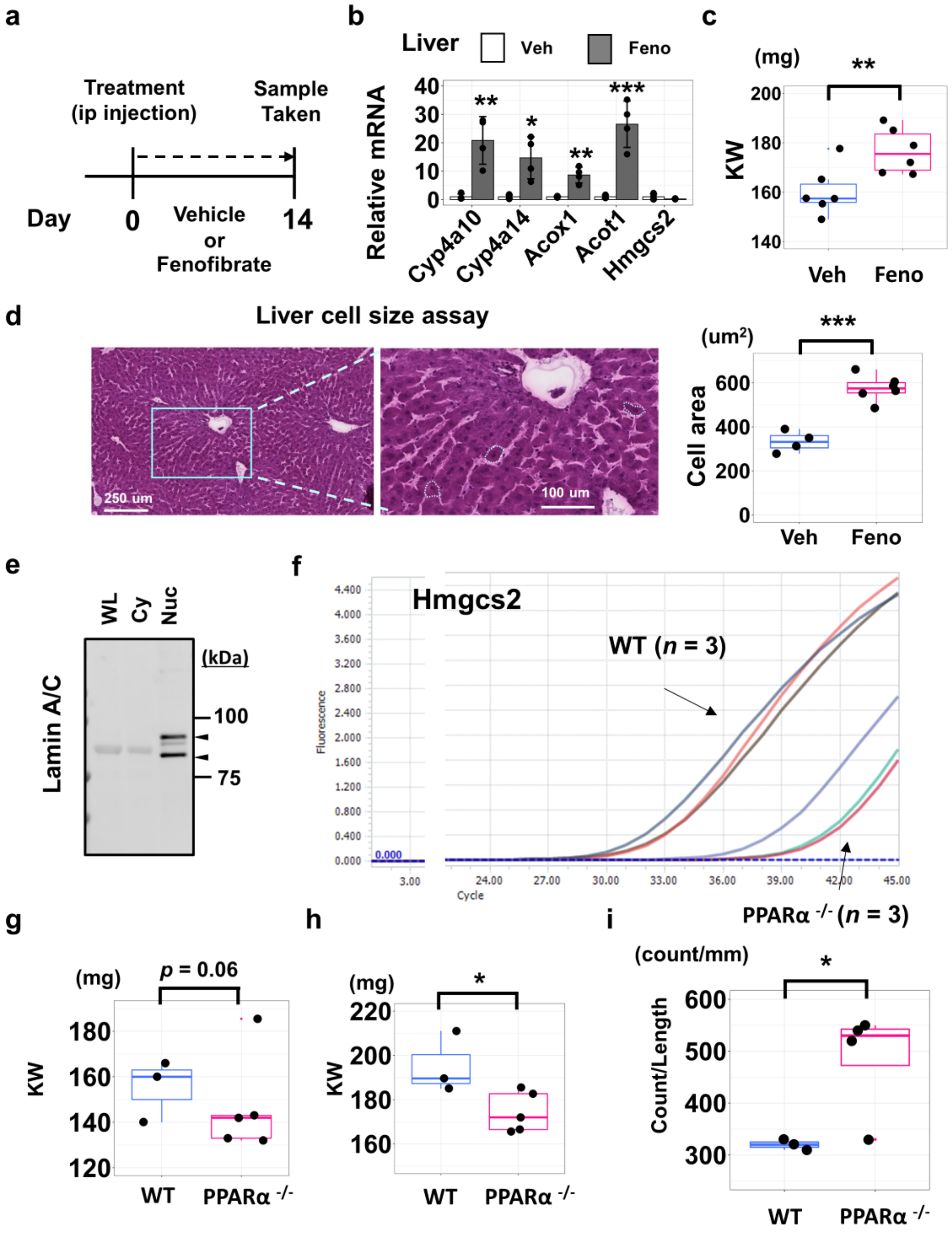

Supplemental Figure 7. Evaluation of involvement of PPARα in compensatory renal hypertrophy

(a) Experimental design. Vehicle or fenofibrate (50 mg/kg BW) was administered peritoneally for 14 days, prior to sample collection.

(b) Expression of selected PPARα target genes in the liver determined using qRT-PCR. Prior to sample collection, mice were treated with either fenofibrate (Feno, grey) or vehicle (Veh, white). Data are the average of four biological replicates. Data were normalized using $2^{(-\Delta\Delta Ct)}$ method. Data are presented as mean ± SD. (*n* = 4 for each group) *$p < 0.05$, **$p < 0.01$, ***$p < 0.001$, unpaired, two-sided student T- test. (Cyp4a10: p = 0.003, Cyp4a14: p = 0.0101, Acox1: p = 0.0016, Acot1: p = 0.00079, Hmgcs2: p = 0.184)

(c) Kidney weight (KW) was significantly increased in the mice treated with fenofibrate (Feno) relative to the mice treated with vehicle (Veh) for 14 days. (*n* = 6 for each group). **$p < 0.01$. (p = 0.004, unpaired, two-sided student t- test).

(d) Liver cell size (area) assay using H-E stained liver from mice with vehicle or fenofibrate. (*n* = 4 for vehicle (Veh), *n* = 6 for fenofibrate (Feno, Grey). ***$p < 0.001$. (p = 0.00013, unpaired, two-sided student t- test).

(e) Western blot of Lamin A/C confirming the enrichment of nuclear fraction. Arrow indicates the bands at the expected molecular weight of Lamin A/C.

(f) Example of quantitative fluorescent signal curves comparing Hmgcs2 gene expression levels between WT and PPARα-null mice. $n = 3$ for each group.

(g) Kidney weight (KW) in WT and PPARα-null mice for the resected left kidney at UNx surgery. KW were decreased (not statistically significant) in the PPARα-null mice relative to the WT mice. $n = 3$ for WT, $n = 5$ for PPARα-null. (p= 0.061, unpaired, two-sided student T- test).

(h) Kidney weight (KW) in WT and PPARα-null mice for the remnant kidney 3 days after UNx surgery mice. Significant changes were found between WT and PPARα-null mice. $n = 3$ for WT, $n = 5$ for PPARα-null. *$p < 0.05$. (p = 0.041, unpaired, two-sided student T- test)

(i) Cell count per unit length was significantly increased in PPARα-null mice. $n = 3$ for WT, $n = 4$ for PPARα-null. *$p < 0.05$. (p = 0.039, unpaired, two-sided student T- test)

Box-and-whisker plots represent mean and 25th and 75th percentiles—interquartile range; IQR—and whiskers extend to maximum and minimum values.

a

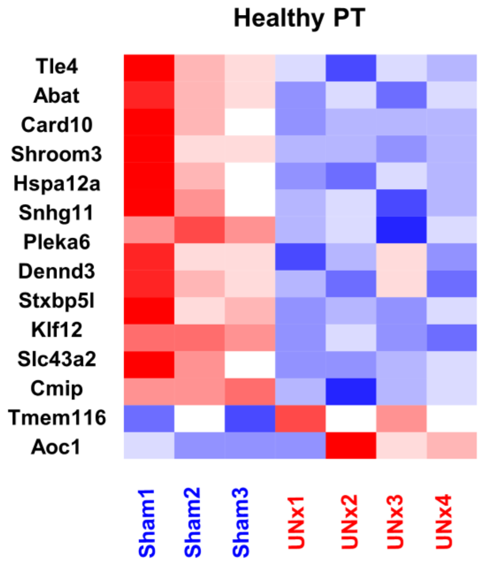

b

Cluster 1 (Healthy S1)				
	Hypertrophy UNx		IRI (PMID: 31506348)	
external_gene_name	Padj	Log (UNx/sham)	PCT.1	P-value_adj
Aoc1	0.02	1.61	0.133	0.00
Tmem116	0.04	0.83	0.179	0.00
Slc22a28	0.06	0.76	0.288	0.00
Slc5a11	0.03	0.73	0.173	0.00
Cldn10	0.09	0.62	0.165	0.00
Klf12	0.08	-0.65	0.249	0.00
Slc43a2	0.07	-0.66	0.109	0.00
Cmip	0.08	-0.68	0.590	0.00
Plekha6	0.08	-0.72	0.114	0.00
Dpyd	0.07	-0.74	0.350	0.00
Card10	0.03	-0.77	0.128	0.00
Dennd3	0.02	-0.82	0.139	0.00
Stxbp5l	0.01	-0.87	0.251	0.00
Abat	0.06	-0.88	0.196	0.00
Hspa12a	0.04	-0.89	0.132	0.00
Shroom3	0.03	-0.92	0.208	0.00
Tle4	0.03	-0.92	0.127	0.00
Snhg11	0.00	-1.01	0.430	0.00

c

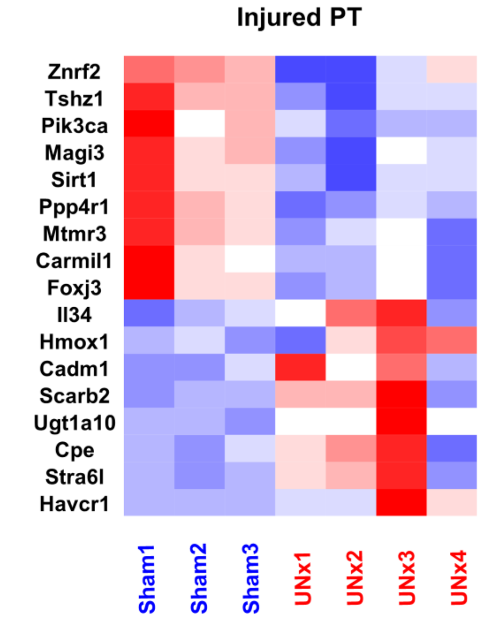

d

Cluster 5 +7 (Injured PT)				
	Hypertrophy UNx		Acute Renal Injury (PMID: 31506348)	
external_gene_name	Padj	Log (UNx/sham)	PCT.1	P-value_adj
Havcr1	0.00	6.41	0.134	0.00
Stra6l	0.02	3.66	0.141	0.00
Cpe	0.08	3.08	0.137	0.00
Ugt1a10	0.08	2.07	0.165	0.00
Scarb2	0.07	1.86	0.104	0.00
Cadm1	0.03	1.03	0.349	0.00
Hmox1	0.03	1.00	0.118	0.00
Il34	0.04	0.82	0.448	0.00
Mtmr3	0.09	-0.67	0.319	0.00
Ppp4r1	0.06	-0.69	0.224	0.00
Foxj3	0.04	-0.73	0.102	0.00
Carmil1	0.07	-0.78	0.331	0.00
Pik3ca	0.07	-0.88	0.109	0.00
Sirt1	0.08	-0.89	0.105	0.00
Magi3	0.03	-1.00	0.174	0.00
Znrf2	0.01	-1.17	0.232	0.00
Tshz1	0.00	-1.24	0.125	0.00

Supplemental Figure 8. Deconvolution analysis using mouse IRI snRNA-seq data [1]

(a) Heatmap for normalized RNA-seq data labelled as "Healthy PT" in the single cell RNA seq study using mouse ischemic renal injury model [1](Log_{10} transformed, scaled and centered) from proximal tubule of Sham (*n* = 3) and UNx (*n* = 4) at the 24 h timepoint. Heatmap show the differentially expressed genes (P_{adj}<0.1) annotated as "Healthy PT" in the previous study.

(b) Data table for RNA-seq data annotated as "Healthy PT" in the single cell RNA seq study using mouse ischemic renal injury model. FDR adjusted *p* value (Benjamini and Hochberg method) and Log_2 (UNx/Sham) in this study, and values of PCT.1 (fraction of cells expressing the genes in the selected cells) and FDR adjusted p value (calculated based on Wilcoxon rank sum test for single cell gene expression (from Seurat package)) in mouse ischemic renal injury (IRI) scRNA-seq data are shown.

(c) Heatmap for normalized RNA-seq data annotated as "Injured PT" in the single cell RNA seq study using mouse ischemic renal injury model (Log_{10} transformed, scaled and centered) from proximal tubule of Sham (*n* = 3) and UNx (*n* = 4) at the 24 h timepoint. Heatmap show the differentially expressed genes (P_{adj}<0.1) annotated as "Injured PT" in the previous study.

(d) Data table for RNA-seq data annotated as "Injured PT" in the single cell RNA seq study using mouse ischemic renal injury model. FDR adjusted *p* value (Benjamini and Hochberg method) and Log_2 (UNx/Sham) in this study, and values of PCT.1 (fraction of cells expressing

the genes in the selected cells) and FDR adjusted *p* value (calculated based on Wilcoxon rank sum test for single cell gene expression (from Seurat package)) in mouse ischemic renal injury (IRI) scRNA-seq data are shown.

Supplementary Tables

Supplementary Table 1. Top 20 abundant S1 proximal tubule transcription factors and hypothetical signal transduction mechanisms in compensatory hypertrophy

Official gene symbol	Annotation	mRNA abundance (TPM)[&]	Rank (of 1273)	Transduction pathway*
Id2	DNA-binding protein inhibitor ID-2	539.0	1	Shear Forces; TGF-b
Ybx1	Y-box-binding protein 1	316.4	2	Ocln-ZO1-ZONAB
Nr1h4	farnesoid X-activated receptor (FXR)	134.4	6	Lipid-Sensing Nuclear Receptors
Hnf4a	hepatocyte nuclear factor 4-alpha	76.4	21	Lipid-Sensing Nuclear Receptors
Ppara	peroxisome proliferator-activated receptor alpha	60.2	29	Lipid-Sensing Nuclear Receptors
Nr1h2	oxysterols receptor LXR-beta (LXR-b)	48.1	37	Lipid-Sensing Nuclear Receptors
Nr1h3	oxysterols receptor LXR-alpha (LXR-a)	48.0	39	Lipid-Sensing Nuclear Receptors
Srebf2	sterol regulatory element-binding protein 2	36.3	47	Cadherin-AMPK
Jund	transcription factor Jun-D	36.1	48	Shear Forces; MAP kinase<br>Growth Hormone-IGF1
Smad4	mothers against decapentaplegic homolog 4	33.7	53	Shear Forces; TGF-b
Nr3c1	glucocorticoid receptor	31.4	58	Glucocorticoid
Stat6	signal transducer and transcription activator 6	29.0	68	Shear Forces; JAK-Stat<br>Growth Hormone-GHR
Stat1	signal transducer and activator of transcription 1	26.2	78	Shear Forces; JAK-Stat<br>Growth Hormone-GHR
Jun	transcription factor AP-1	23.5	95	Shear Forces; MAP kinase<br>Growth Hormone-IGF1
Etv1	ETS translocation variant 1	21.9	100	Shear Forces; MAP kinase<br>Growth Hormone-IGF1
Crem	cAMP-responsive element modulator	21.7	102	ANP-cGMP-PKG<br>Beta-adrenergic-cAMP-PKA
Stat3	signal transducer and activator of transcription 3	20.9	107	Growth Hormone-GHR<br>Shear Forces; MAP kinase
Smad2	SMAD family member 2	15.7	151	Shear Forces; TGF-b
E2f4	E2F transcription factor 4	15.3	156	Integrin
Srebf1	sterol regulatory element-binding transcription factor 1	13.0	183	Cadherin-AMPK

& Single tubule RNA-seq data for S1 proximal tubule from Chen et al. (PMID:33769951)

*** Hypothetical pathways for hypertrophic response to unilateral nephrectomy (Supplementary Data 4)**

Supplementary Table 2. Top ranking of Hallmark gene set enrichment analysis (GSEA) of genes changed in abundance in right kidney after unilateral nephrectomy of left kidney (versus Sham nephrectomy) in microdissected proximal tubules

Hallmark Gene Sets	Time after surgery	Direction of Change	Normalized Enrichment Score (NES)	FDR	Top Genes selected
FATTY ACID METABOLISM	24 hr	up	2.28	< 0.00001	Fabp1, Vnn1, Hmgcs2, Mgll, Ehhadh, Cd36, Acot2, Acadl
MTORC1_SIGNALING	24 hr	up	2.01	4.1E-04	Sec11a, Ebp, Cdkn1a, Ddx39a, Cd9, Ctsc
ADIPOGENESIS	24 hr	up	1.99	3.1E-04	Ucp2, Angptl4, Mgll, Cd36, Acadl, Cpt2
MITOTIC_SPINDLE	24 hr	down	-1.99	3.1E-03	Ranbp9, Pxn, Numa1, Cep72, Itsn1, Septin9
E2F_TARGETS	72 hr	up	1.85	< 0.00001	Birc5, Top2a, Cdk1, Rem2, Cdkn3, Plk1, Mcm5, Aurka
G2M_CHECKPOINT	72 hr	up	1.84	< 0.00001	Knl1, Birc5, Top2a, Pbk, Cdk1
OXIDATIVE_PHOSPHORYLATION	72 hr	up	1.72	1.3E-02	not available
INTERFERON_ALPHA_RESPONSE	72 hr	down	-1.51	9.0E-02	not available

Supplementary Table 3. Top ranking of Hallmark gene set enrichment analysis (GSEA) of genes changed in abundance in the right kidney after unilateral nephrectomy of the left kidney (versus Sham nephrectomy) in microdissected cortical collecting ducts

Hallmark Gene Sets	Time after surgery	Direction of Change	Normalized Enrichment Score (NES)	FDR	Top Genes selected
E2F_TARGETS	24 hr	up	2.20	< 0.00001	Birc4, Plk1, Ccnb2, Mxd3, Melk, Stmn1, Cdkn3, Cdk1
G2M_CHECKPOINT	24 hr	up	2.05	< 0.00001	Birc5, Ube2c, Plk1, Pbk, Ccnb2, Ndc80, Tpx2
MYC_TARGETS_V1	24 hr	up	1.73	8.0E-03	not available
MITOTIC_SPINDLE	24 hr	up	1.63	4.0E-02	not available
GLYCOLYSIS	24 hr	up	1.62	4.7E-02	not available
KRAS_SIGNALING_UP	24 hr	down	-1.80	1.0E-01	not available
INFLAMMATORY_RESPONSE	24 hr	down	-1.84	4.7E-02	Ccl20, Kif1b, Abca1, Cscl10, Nfkbia
TNFA_SIGNALING_VIA_NFKB	24 hr	down	-1.91	1.6E-02	Ccl20, Ccnd1, Cxcl1, Nfat5, Cscl2, Ccl4, Abca1
OXIDATIVE_PHOSPHORYLATION	72 hr	up	1.59	0.001	not available
E2F_TARGETS	72 hr	up	1.51	0.011	not available
G2M_CHECKPOINT	72 hr	up	1.47	0.038	not available

Supplementary Table 4. Sequences of oligonucleotide primers used for qRT-PCR

Gene name	Forward	Reverse
Beta actin	TCCTTCTTGGGTATGGAATCCTG	AGCTCAGTAACAGTCCGCCTA
Cyp4a10	AAGGGTCAAACACCTCTGGA	GATGGACGCTCTTTACCCAA
Cyp4a14	AGCAAACTGTTTCCCAATGC	ACCCCTCTAGATTTGCACCA
Acox1	GGGCACGGCTATTCTCACAG	CATCAAGAACCTGGCCGTCT
Acot1	CGATGACCTCCCCAAGAACAT	CTTTTACCTCGGGGTGGCT
Hmgcs2	AGAAATCCCTGGCTCGGTTG	AGCTTTAGACCCCTGAAGGC

Supplementary Notes

Supplementary Data 1. Raw data of kidney weight and body weight in mice without (Sham) or with unilateral nephrectomy (UNx) at different time points (Days 1, Day 3, and 30).

Supplementary Data 2. Histological analysis of kidney from mice without (Sham) or with unilateral nephrectomy (UNx) surgery at the 72 hour timepoint.

Supplementary Data 3. Morphological data of IF-stained microdissected S1 proximal tubules and cortical collecting duct (CCD) 30 days after surgery.

Supplementary Data 4. The hypothesis signaling pathways in kidney triggered by unilateral nephrectomy.

Supplementary Data 5. The enriched TF binding motifs identified by HOMER de novo motif analysis in chromatin regions that are more accessible in UNx (DAR-UP), those that are less accessible in UNx (DAR-down), and all chromatin regions that are identified in S1 segment of the proximal tubule.

Supplementary Data 6. A quantitative comparison of chromatin accessibility in microdissected S1 segment of proximal tubules at 24 h after UNx.

Supplementary Data 7. Target gene sets for individual transcription factors listed in **Supplementary Table 1.**

Supplementary Data 8. Summary of transcription factor target gene sets analysis for ATAC-seq data (24h proximal tubule), RNA-seq data for proximal tubules at 24 h and 72 h after surgery.

Supplementary Data 9. Transcript abundance changes in microdissected S1 segment of PTs of mice at 24 h after UNx.

Supplementary Data 10. Upstream regulator analysis using QIAGEN's Ingenuity Pathway Analysis for S1 proximal tubule RNA-seq dataset at the 24h timepoint.

Supplementary Data 11. Transcript abundance changes in microdissected CCDs of mice at 24 h after UNx.

Supplementary Data 12. Transcript abundance changes in microdissected CCDs of mice at 72 h after UNx.

Supplementary Data 13. Transcript abundance changes in microdissected S1 segment of PTs of mice at 72 h after UNx.

Supplementary Data 14. Comparison of the core enrichments of genes annotated as either "G2M CHECKPOINT" or "E2F_TARGET" between S1 proximal tubule RNA seq at the 72 hour time point and CCD RNA seq at the 24 hour timepoint.

Supplementary Data 15. Upstream regulator analysis using QIAGEN's Ingenuity Pathway Analysis for S1 proximal tubule RNA-seq dataset at the 72 h timepoint.

Supplementary Data 16. Protein abundance changes in whole kidney of mice at 24 h after UNx.

Supplementary Data 17. Upstream regulator analysis using QIAGEN's Ingenuity Pathway Analysis for whole kidney proteomics dataset at the 24 h timepoint.

Supplementary Data 18. Protein abundance changes in kidney cortex of mice at 24 h after UNx.

Supplementary Data 19. Upstream regulator analysis using QIAGEN's Ingenuity Pathway Analysis for kidney cortex proteomics dataset at the 24 h timepoint.

Supplementary Data 20. Protein abundance changes in whole kidney of mice at 72 h after UNx.

Supplementary Data 21. Upstream regulator analysis using QIAGEN's Ingenuity Pathway Analysis for whole kidney proteomics dataset at the 72 h timepoint.

Supplementary Data 22. Phosphoprotein abundance changes in whole kidney of mice at 24 h after UNx.

Supplementary Data 23. Phosphoprotein abundance changes in whole kidney of mice at 72 h after UNx.

Supplementary Data 24. Protein kinases that underwent changes in phosphorylation in contralateral kidney in response to unilateral nephrectomy (UNx) relative to Sham surgery at 72 h.

Supplementary Data 25. Data integration analysis using multi-omics datasets.

Supplementary Data 26. The changes in abundance of amino acid transporters in transcriptome and proteome.

Supplementary Data 27. Fatty acid (Gas chromatography) and lipid analysis (calorimetric assay) of kidney from Sham or UNx at 24 hour after surgery.

Supplementary Data 28. Body weight, kidney weight and histological parameters of the kidney from mice with or without fenofibrate treatments for 14 days.

Supplementary Data 29. Morphological data of IF-stained microdissected S1 proximal tubules from mice without (vehicle) and with fenofibrate treatments for 14 days.

Supplementary Data 30. Cell size assay for mouse livers with our without fenofibrates treatments for 14 days using NDP Nanozoomer.

Supplementary Data 31. Morphological data of IF-stained microdissected S1 proximal tubules from WT mice or PPARα $^{-/-}$ mice 3 days after unilateral nephrectomy.

Supplementary Discussion 1

Hypothesis Group 1. Nutrient-mediated signaling including the "mechanistic target of rapamycin" (mTOR) pathway, a classic growth pathway[2,3], and the "peroxisome proliferator-activated receptor alpha" (PPARα)/ "hepatocyte nuclear factor 4-alpha"(HNF4α) signaling pathway. (Increased GFR increases the amount of amino acids and fatty acids filtered resulting in increased amino acid/ fatty acid transport across the apical plasma membrane of proximal tubule cells with concomitant increases in free amino acid/ fatty acid concentration in the cells.) **Hypothesis Group 2.** Increased bending of the primary cilium due to accelerated luminal flow secondary to increased GFR, resulting in alteration of Hedgehog signaling, cAMP-PKA signaling and calcium mobilization[4-7]. **Hypothesis Group 3**. Increased luminal pressure flattens the proximal tubule epithelium[8] altering forces at cell-cell or cell-extracellular matrix contact points which are transduced into intracellular signals. This would potentially include the Hippo pathway (instrumental in organ size control[9,10]), the Wnt pathway[11], tight junction associated signals including AMP-stimulated kinase signaling[12], integrin-related signaling, and growth factor signaling via ephrin receptors[13]. Stretch activated calcium channels like Piezo1 and Piezo2 could also mediate changes in gene expression through increases in intracellular calcium[14]. **Hypothesis Group 4.** Increased proximal tubule re-absorptive rate secondary to increased GFR could result in signaling changes owing to associated increases in ATP utilization

for transport. This increase in ATP utilization would potentially result in localized decreases in ATP availability, essentially competing with other ATP dependent processes including protein phosphorylation and cAMP generation. In addition, SNF1-family protein kinases including AMPK kinase can be regulated though changes in adenine nucleotide abundances[15]. **Hypothesis Group 5.** Increased flow rate of proximal tubule secondary to increased single nephron GFR could result in signaling changes related to shear force[16]. This would potentially include the TGF-beta, MAPK, Wnt and JAK-STAT signaling[16]. **Hypothesis Group 6.** The acute reduction in GFR can result in increased extracellular fluid (ECF) volume that can suppress the renin-angiotensin-aldosterone pathway that could be involved in the overall hypertrophic response of the kidney[17]. Also, increased ECF may be related to increased atrial natriuretic peptide (ANP) secretion by the heart and decreased release of norepinephrine[18,19]. **Hypothesis Group 7.** Unilateral nephrectomy may result in accumulation of insulin, the growth hormone (GH)/insulin-like growth factor-1 (IGF-1) followed by activation of this pathway[20-22] or cause stress-induced increases in corticosteroid secretion[23] which can contribute to transcriptional changes after unilateral nephrectomy.

Supplementary Discussion 2

To determine whether the proximal tubule status induced by UNx is relevant to post-acute renal injury model, we performed deconvolution analysis for our PT-S1 RNA seq dataset at 24 hours on published mouse ischemic renal injury (IRI) snRNA-seq datasets[1] and looked for any change in cell-type proportions consistent with compensatory hypertrophy (**Supplementary Fig. 8**). From this analysis, we can see some pattern showing that "Healthy-PT" related genes tend to be decreased in UNx kidney (**Supplementary Fig. 8A**), and "Injured-PT" related genes are both increased and decreased in UNx kidney (**Supplementary Fig. 8B**). Notably, however, most differentially expressed genes (p < 0.1) in our study annotated as either "Healthy PT" or "Injured PT" have small PCT.1 values (fraction of cells expressing the genes in the selected cells) defined by the mouse IRI snRNA-seq study (**Supplementary Fig. 8C** and **D**), indicating difficulty in associating our renal hypertrophy model with this acute renal injury model due to sensitivity. Future studies would benefit from deconvolution analysis of our study on single cell RNA-seq using other kidney disease models in which PPARα activation has been reported to be relevant previously[24-26].

Supplementary References

1. Wilson, P.C.*, et al.* The single-cell transcriptomic landscape of early human diabetic nephropathy. *Proc Natl Acad Sci U S A* **116**, 19619-19625 (2019).

2. Chen, J.K., Chen, J., Neilson, E.G. & Harris, R.C. Role of mammalian target of rapamycin signaling in compensatory renal hypertrophy. *J Am Soc Nephrol* **16**, 1384-1391 (2005).

3. Chen, J.K., Chen, J., Thomas, G., Kozma, S.C. & Harris, R.C. S6 kinase 1 knockout inhibits uninephrectomy- or diabetes-induced renal hypertrophy. *Am J Physiol Renal Physiol* **297**, F585-593 (2009).

4. Wheway, G., Nazlamova, L. & Hancock, J.T. Signaling through the Primary Cilium. *Front Cell Dev Biol* **6**, 8 (2018).

5. Winyard, P. & Jenkins, D. Putative roles of cilia in polycystic kidney disease. *Biochim Biophys Acta* **1812**, 1256-1262 (2011).

6. Praetorius, H.A. & Spring, K.R. Bending the MDCK cell primary cilium increases intracellular calcium. *J Membr Biol* **184**, 71-79 (2001).

7. Tschaikner, P., Enzler, F., Torres-Quesada, O., Aanstad, P. & Stefan, E. Hedgehog and Gpr161: Regulating cAMP Signaling in the Primary Cilium. *Cells* **9**(2020).

8. Maunsbach, A.B. & Boulpaep, E.L. Hydrostatic pressure changes related to paracellular shunt ultrastructure in proximal tubule. *Kidney Int* **17**, 732-748 (1980).

9. Yu, F.X., Zhao, B. & Guan, K.L. Hippo Pathway in Organ Size Control, Tissue Homeostasis, and Cancer. *Cell* **163**, 811-828 (2015).

10. Moya, I.M. & Halder, G. Hippo-YAP/TAZ signalling in organ regeneration and regenerative medicine. *Nat Rev Mol Cell Biol* **20**, 211-226 (2019).

11. Bienz, M. beta-Catenin: a pivot between cell adhesion and Wnt signalling. *Curr Biol* **15**, R64-67 (2005).

12. Isogai, T., Park, J.S. & Danuser, G. Cell forces meet cell metabolism. *Nat Cell Biol* **19**, 591-593 (2017).

13. Vreeken, D., Zhang, H., van Zonneveld, A.J. & van Gils, J.M. Ephs and Ephrins in Adult Endothelial Biology. *Int J Mol Sci* **21**(2020).

14. Kefauver, J.M., Ward, A.B. & Patapoutian, A. Discoveries in structure and physiology of mechanically activated ion channels. *Nature* **587**, 567-576 (2020).

15. Kikuchi, H., *et al.* Failure to sense energy depletion may be a novel therapeutic target in chronic kidney disease. *Kidney Int* **95**, 123-137 (2019).

16. Kunnen, S.J., Malas, T.B., Semeins, C.M., Bakker, A.D. & Peters, D.J.M. Comprehensive transcriptome analysis of fluid shear stress altered gene expression in renal epithelial cells. *J Cell Physiol* **233**, 3615-3628 (2018).

17. Yang, T. & Xu, C. Physiology and Pathophysiology of the Intrarenal Renin-Angiotensin System: An Update. *J Am Soc Nephrol* **28**, 1040-1049 (2017).

18. Gardner, D.G., Chen, S., Glenn, D.J. & Grigsby, C.L. Molecular biology of the natriuretic peptide system: implications for physiology and hypertension. *Hypertension* **49**, 419-426 (2007).

19. Rauch, A.L. & Campbell, W.G., Jr. Nephrectomy-induced alterations in the synthesis of catecholamines in the sympathetic nervous system and central nervous system. *Am J Hypertens* **1**, 50-53 (1988).

20. Kato, Y., *et al.* Divergent effects of unilateral and subtotal nephrectomy on insulin sensitivity in rats. *Ren Fail* **27**, 451-457 (2005).

21. Rabkin, R. & Schaefer, F. New concepts: growth hormone, insulin-like growth factor-I and the kidney. *Growth Horm IGF Res* **14**, 270-276 (2004).

22. Haffner, D., Grund, A. & Leifheit-Nestler, M. Renal effects of growth hormone in health and in kidney disease. *Pediatr Nephrol* **36**, 2511-2530 (2021).

23. Greenwood, F.C. & Landon, J. Growth hormone secretion in response to stress in man. *Nature* **210**, 540-541 (1966).

24. Li, S.*, et al.* Transgenic expression of proximal tubule peroxisome proliferator-activated receptor-alpha in mice confers protection during acute kidney injury. *Kidney Int* **76**, 1049-1062 (2009).

25. Li, S.*, et al.* PPAR alpha ligand protects during cisplatin-induced acute renal failure by preventing inhibition of renal FAO and PDC activity. *Am J Physiol Renal Physiol* **286**, F572-580 (2004).

26. Nagothu, K.K., Bhatt, R., Kaushal, G.P. & Portilla, D. Fibrate prevents cisplatin-induced proximal tubule cell death. *Kidney Int* **68**, 2680-2693 (2005).
