# Supplementary material for: Signaling mechanisms in renal compensatory hypertrophy revealed by multi-omics
Source: Nat Commun. 2023 Jun 16;14:3481. doi: 10.1038/s41467-023-38958-9 (PMC10276015; doi:10.1038/s41467-023-38958-9)
Supplement: Supplementary file 3 — Description of Additional Supplementary Files [file 41467_2023_38958_MOESM3_ESM.pdf]

Description of Additional Supplementary Files

Supplementary Data 1. Raw data of kidney weight and body weight in mice without (Sham) or with unilateral nephrectomy (UNx) at different time points (Days 1, Day 3, and 30).

Supplementary Data 2. Histological analysis of kidney from mice without (Sham) or with unilateral nephrectomy (UNx) surgery at the 72 hour timepoint.

Supplementary Data 3. Morphological data of IF-stained microdissected S1 proximal tubules and cortical collecting duct (CCD) 30 days after surgery.

Supplementary Data 4. The hypothesis signaling pathways in kidney triggered by unilateral nephrectomy.

Supplementary Data 5. The enriched TF binding motifs identified by HOMER de novo motif analysis in chromatin regions that are more accessible in UNx (DAR-UP), those that are less accessible in UNx (DAR-down), and all chromatin regions that are identified in S1 segment of the proximal tubule.

Supplementary Data 6. A quantitative comparison of chromatin accessibility in microdissected S1 segment of proximal tubules at 24 h after UNx.

Supplementary Data 7. Target gene sets for individual transcription factors listed in **Supplementary Table 1.**

Supplementary Data 8. Summary of transcription factor target gene sets analysis for ATAC-seq data (24h proximal tubule), RNA-seq data for proximal tubules at 24 h and 72 h after surgery.

Supplementary Data 9. Transcript abundance changes in microdissected S1 segment of PTs of mice at 24 h after UNx.

Supplementary Data 10. Upstream regulator analysis using QIAGEN's Ingenuity Pathway Analysis for S1 proximal tubule RNA-seq dataset at the 24h timepoint.

Supplementary Data 11. Transcript abundance changes in microdissected CCDs of mice at 24 h after UNx.

Supplementary Data 12. Transcript abundance changes in microdissected CCDs of mice at 72 h after UNx.

Supplementary Data 13. Transcript abundance changes in microdissected S1 segment of PTs of mice at 72 h after UNx.

Supplementary Data 14. Comparison of the core enrichments of genes annotated as either "G2M CHECKPOINT" or "E2F_TARGET" between S1 proximal tubule RNA seq at the 72 hour time point and CCD RNA seq at the 24 hour timepoint.

Supplementary Data 15. Upstream regulator analysis using QIAGEN's Ingenuity Pathway Analysis for S1 proximal tubule RNA-seq dataset at the 72 h timepoint.

Supplementary Data 16. Protein abundance changes in whole kidney of mice at 24 h after UNx.

Supplementary Data 17. Upstream regulator analysis using QIAGEN's Ingenuity Pathway Analysis for whole kidney proteomics dataset at the 24 h timepoint.

Supplementary Data 18. Protein abundance changes in kidney cortex of mice at 24 h after UNx.

Supplementary Data 19. Upstream regulator analysis using QIAGEN's Ingenuity Pathway Analysis for kidney cortex proteomics dataset at the 24 h timepoint.

Supplementary Data 20. Protein abundance changes in whole kidney of mice at 72 h after UNx.

Supplementary Data 21. Upstream regulator analysis using QIAGEN's Ingenuity Pathway Analysis for whole kidney proteomics dataset at the 72 h timepoint.

Supplementary Data 22. Phosphoprotein abundance changes in whole kidney of mice at 24 h after UNx.

Supplementary Data 23. Phosphoprotein abundance changes in whole kidney of mice at 72 h after UNx.

Supplementary Data 24. Protein kinases that underwent changes in phosphorylation in contralateral kidney in response to unilateral nephrectomy (UNx) relative to Sham surgery at 72 h.

Supplementary Data 25. Data integration analysis using multi-omics datasets.

Supplementary Data 26. The changes in abundance of amino acid transporters in transcriptome and proteome.

Supplementary Data 27. Fatty acid (Gas chromatography) and lipid analysis (calorimetric assay) of kidney from Sham or UNx at 24 hour after surgery.

Supplementary Data 28. Body weight, kidney weight and histological parameters of the kidney from mice with or without fenofibrate treatments for 14 days.

Supplementary Data 29. Morphological data of IF-stained microdissected S1 proximal tubules from mice without (vehicle) and with fenofibrate treatments for 14 days.

Supplementary Data 30. Cell size assay for mouse livers with our without fenofibrates treatments for 14 days using NDP Nanozoomer.

Supplementary Data 31. Morphological data of IF-stained microdissected S1 proximal tubules from WT mice or PPARα $^{-/-}$ mice 3 days after unilateral nephrectomy.
