# Supplementary material for: Signaling mechanisms in renal compensatory hypertrophy revealed by multi-omics
Source: Nat Commun. 2023 Jun 16;14:3481. doi: 10.1038/s41467-023-38958-9 (PMC10276015; doi:10.1038/s41467-023-38958-9)
Supplement: Supplementary file 35 — Reporting Summary [file 41467_2023_38958_MOESM35_ESM.pdf]

nature portfolio

Corresponding author(s): Hiroaki Kikuchi

Last updated by author(s): 2023/04/15

Reporting Summary

Nature Portfolio wishes to improve the reproducibility of the work that we publish. This form provides structure for consistency and transparency in reporting. For further information on Nature Portfolio policies, see our Editorial Policies and the Editorial Policy Checklist.

Statistics

For all statistical analyses, confirm that the following items are present in the figure legend, table legend, main text, or Methods section.

n/a	Confirmed	
☐	☒	The exact sample size (*n*) for each experimental group/condition, given as a discrete number and unit of measurement
☐	☒	A statement on whether measurements were taken from distinct samples or whether the same sample was measured repeatedly
☐	☒	The statistical test(s) used AND whether they are one- or two-sided<br>*Only common tests should be described solely by name; describe more complex techniques in the Methods section.*
☐	☒	A description of all covariates tested
☐	☒	A description of any assumptions or corrections, such as tests of normality and adjustment for multiple comparisons
☐	☒	A full description of the statistical parameters including central tendency (e.g. means) or other basic estimates (e.g. regression coefficient) AND variation (e.g. standard deviation) or associated estimates of uncertainty (e.g. confidence intervals)
☐	☒	For null hypothesis testing, the test statistic (e.g. *F*, *t*, *r*) with confidence intervals, effect sizes, degrees of freedom and *P* value noted<br>*Give P values as exact values whenever suitable.*
☒	☐	For Bayesian analysis, information on the choice of priors and Markov chain Monte Carlo settings
☒	☐	For hierarchical and complex designs, identification of the appropriate level for tests and full reporting of outcomes
☒	☐	Estimates of effect sizes (e.g. Cohen's *d*, Pearson's *r*), indicating how they were calculated

Our web collection on statistics for biologists contains articles on many of the points above.

Software and code

Policy information about availability of computer code

Data collection	NDP Nanozoomer HT (Hamamatsu Photonics), ZENBlue software (Zeiss), MaxQuant 1.6.17.0 (Max Planck Institute of Biochemistry), a LightCycler® 96 System (Roche), Odyssey CLx Imaging System (LI-COR Biosciences)
Data analysis	Statistical analysis was performed in R 4.2 and SPSS 17.0 statistical software (SPSS, Inc., Chicago, IL, USA).<br>Custom code is available via https://esbl.nhlbi.nih.gov/Databases/UNx-Supp/.<br><br># RNA-seq<br>RNA-seq reads were indexed using STAR (2.7.6a) and aligned to the mouse reference genome from Ensembl (release 103) using STAR (2.7.6a).<br>Read quality was assessed using fastQC (0.11.9).<br>Transcripts per million (TPM) and expected read counts were generated using RSEM (1.3.3).<br>Differential expression was assessed using R packages edgeR (v3.40.2) and DESeq2 (v1.39.8).<br><br># ATAC-seq<br>Adapter sequences were trimmed for both forward and reverse reads using cutadapt (3.4).<br>Read quality was assessed using fastQC (0.11.9).<br>Trimmed sequences were aligned to the mouse reference genome mm10 using bowtie2 (2.4.4).<br>The resulting SAM files were converted to a binary format (BAM), sorted and indexed using samtools (v 1.14)<br>Identically mapping read duplicates were marked using Picard (v 2.25.7) MarkDuplicates (Broad Institute. Picard toolkit. Broad Institute, GitHub repository (2019)).<br>BAM files were converted to BED format using BedTools (v2.30.0).

Narrow open chromatin peaks were identified using MACS2 (v 2.2.7.1)
Reads mapping to ENCODE blacklist regions66 were discarded using BedTools (v 2.30.0).
bigwig files were generated using bamCoverage.
Read distribution was visualized on the UCSC genome browser.
Differentially accessible regions (DAR) were identified using R package DiffBind (v 3.8.4).
The footprint analysis was done by the factorFootprints function under the R package ATACseqQC (v 1.22.0).

Image J (NIH), ZENBlue software (Zeiss), IMARIS Scientific Image Processing & Analysis software (v7.7.1, Bitplane, Zurich), IPA (Qiagen), MaxQuant 1.6.17.0 (Max Planck Institute of Biochemistry), GSEA (http://software.broadinstitute.org/gsea/index.jsp)

For manuscripts utilizing custom algorithms or software that are central to the research but not yet described in published literature, software must be made available to editors and reviewers. We strongly encourage code deposition in a community repository (e.g. GitHub). See the Nature Portfolio guidelines for submitting code & software for further information.

Data

Policy information about availability of data

All manuscripts must include a data availability statement. This statement should provide the following information, where applicable:

- Accession codes, unique identifiers, or web links for publicly available datasets
- A description of any restrictions on data availability
- For clinical datasets or third party data, please ensure that the statement adheres to our policy

Raw fastq files and raw count information from the RNA-seq analysis and ATAC-seq analysis were deposited on the GEO (GSE211021, GSE211022).
https://www.ncbi.nlm.nih.gov/geo/query/acc.cgi?acc=GSE211021
https://www.ncbi.nlm.nih.gov/geo/query/acc.cgi?acc=GSE211022

The proteomics data are deposited to the ProteomeXchange Consortium via the PRIDE partner repository with the data identifier
PXD036395 (whole kidney)
PXD039697 (Kidney cortex)

CHEA Transcription factor targets dataset is available at https://maayanlab.cloud/Harmonizome/dataset/CHEA+Transcription+Factor+Targets

Data Websites
To allow users facile access to the curated data, we have set up a publicly accessible
web resource at
https://esbl.nhlbi.nih.gov/Databases/UnX-proteome/index.html for bulk kidney proteomics (whole kidney),
https://esbl.nhlbi.nih.gov/Databases/UnX-Phospho/72hlog.html for bulk kidney phosphoproteomics.
For mirodissected tubule RNA-seq and ATAC-seq, we have set up Shiny-based web page
https://esbl.nhlbi.nih.gov/UNx/. ATAC-seq data can be also viewable at
https://esbl.nhlbi.nih.gov/IGV_mo/ , in IGV web browser.

Human research participants

Policy information about studies involving human research participants and Sex and Gender in Research.

Reporting on sex and gender	This study does not include human research
Population characteristics	This study does not include human research
Recruitment	This study does not include human research
Ethics oversight	This study does not include human research

Note that full information on the approval of the study protocol must also be provided in the manuscript.

Field-specific reporting

Please select the one below that is the best fit for your research. If you are not sure, read the appropriate sections before making your selection.

[x] Life sciences [ ] Behavioural & social sciences [ ] Ecological, evolutionary & environmental sciences

For a reference copy of the document with all sections, see nature.com/documents/nr-reporting-summary-flat.pdf

Life sciences study design

All studies must disclose on these points even when the disclosure is negative.

Sample size	Sample sizes for omic experiments was determined based on prior experience in similar studies. Note that conclusions are based on ensembles of findings with regard to individual gene products and not on a single gene changing, so in general statistical power is very high (typical of omic experiments)
Data exclusions	Omic data are filtered in an unbiased fashion to remove low abundance/high noise gene products. However, all data are provided in supplementary materials and at data sharing web sites, which allow users to examine and recalculate if desired
Replication	For all data presented in the manuscript, we examined at least three independent biological samples (three different mice) to ensure the reproducibility. All the attemps at replication were successful.
Randomization	Animals were arbitrarily utilized for sham vs uninephrectomy by our surgical core.
Blinding	No blinding was done. One of the main advantages of omic studies is that positive and negative controls are built into the methods since every transcript or protein are measured. Thus, if there was some bias introduced into the study, those negative a positive controls would make it obvious.

Reporting for specific materials, systems and methods

We require information from authors about some types of materials, experimental systems and methods used in many studies. Here, indicate whether each material, system or method listed is relevant to your study. If you are not sure if a list item applies to your research, read the appropriate section before selecting a response.

Materials & experimental systems

n/a	Involved in the study
☐	☒ Antibodies
☒	☐ Eukaryotic cell lines
☒	☐ Palaeontology and archaeology
☐	☒ Animals and other organisms
☒	☐ Clinical data
☒	☐ Dual use research of concern

Methods

n/a	Involved in the study
☒	☐ ChIP-seq
☒	☐ Flow cytometry
☒	☐ MRI-based neuroimaging

Antibodies

Antibodies used

anti-AMPK-alpha (Thr172) antibody (rabbit, Cell signaling # 2535, RRID:AB_331250)
anti-AMPK-α antibody (rabbit, Cell signaling # 2532, RRID:AB_330331)
anti-AKT antibody (rabbit, Cell signaling # 9272, RRID:AB_329827)
anti-AKT (Ser473) antibody (rabbit,Cell signaling # 9271, RRID:AB_329825)
anti-PPARα antibody (rabbit, NOVUS Biologicals # NB600-636)
anti-NaPi-2 antibody (rabbit, in house, #L697)
anti-AQP2 (rabbit, in house, #K5007)
anti-AQP2 (chicken, in house, #CC265)
anti-AQP1 (rabbit, in house, # LL266)
anti Ki-67 (rabbit, Abcam # 16667, RRID:AB_302459)
anti-V-ATPase B1/B2 (mouse, Santa Cruz Biotechnology, #sc-55544)

Alexa Fluor M-594 goat anti-mouse (goat, Invitrogen, #A11032)
Alexa Fluor R-488 goat anti-rabbit (goat, Invitrogen, #A11034)
Alexa Fluor C-488 goat anti-chicken(goat, Invitrogen, #A11039)
Alexa Fluor C-568 goat anti-chicken (goat, Invitrogen, #A11041)

Validation

More detailed information about these antibodies is available on these manufacturer's websites.
Knepper lab antibodies were validated in the original studies. https://pubmed.ncbi.nlm.nih.gov/11678722/

Animals and other research organisms

Policy information about studies involving animals; ARRIVE guidelines recommended for reporting animal research, and Sex and Gender in Research

Laboratory animals	4 to 7 weeks old PPARα wild-type (PPARα+/+) and PPARα-null (PPARα-/-) mice (RRID:IMSR_JAX:008154), both are male C57BL/6 strain, were obtained from Dr. Frank J. Gonzalez in the Laboratory of Metabolism, Center for Cancer Research, National Cancer Institute, National Institutes of Health
Wild animals	Pathogen-free, male, 6-to 8 week-old C57BL/6 mice (Taconic) were used
Reporting on sex	Only adult male mice were used in our experiments. Sex and gender is an important confounding variable in physiological studies along with age and time of day (circadian). Accordingly, all three of these variables (sex, age, and time of day) have been carefully controlled to maximize statistical power with regard to understanding mechanism of renal hypertrophy. In our case, we used only male mice for this reason. Our laboratory currently has other studies focusing on sex and circadian behavior in the renal proximal tubule that are not germane to the main question in our hypertrophy study.
Field-collected samples	This study did not use field-collected samples.
Ethics oversight	All animal experimental procedures were in accordance with National Heart, Lung, and Blood Institute [NHLBI] animal protocol H-0047R6

Note that full information on the approval of the study protocol must also be provided in the manuscript.

March 2021
